# Modified surgical technique for recurrent organized chronic subdural hematoma: a preliminary retrospective case series

**DOI:** 10.3389/fsurg.2026.1850959

**Published:** 2026-06-23

**Authors:** Sheng Li, Guangzhao Li, Yulong Wang, Jianwen Wang, Guanhang Shu, Xiaowang Niu, Xiang Li

**Affiliations:** 1 Department of Neurosurgery, Xinghua People’s Hospital Affiliated to Yangzhou University, Xinghua, China; 2 Department of Neurosurgery, Hefei First People's Hospital, Hefei, China; 3 Department of Gastrointestinal Surgery, Xinghua People's Hospital Affiliated to Yangzhou University, Xinghua, China; 4 Department of Thyroid and Breast Surgery, Xinghua People's Hospital Affiliated to Yangzhou University, Xinghua, China; 5 Department of Neurosurgery, Suqian Hospital Affiliated to Xuzhou Medical University, Suqian, China

**Keywords:** complications, middle meningeal artery occlusion, postoperative recurrence rate, recurrent organized chronic subdural hematoma, temporalis muscle grafting

## Abstract

**Background:**

The incidence of chronic subdural hematoma (CSDH) increases with age, and the recurrence rate after burr-hole drainage remains relatively high. The clinical management of recurrent organized CSDH continues to pose a challenge. This study aimed to preliminarily evaluate the safety, neurological improvement, and recurrence control of craniotomy for hematoma evacuation combined with middle meningeal artery occlusion and temporalis muscle grafting in the treatment of recurrent organized CSDH.

**Methods:**

A retrospective analysis was performed on 17 patients with recurrent organized CSDH admitted between January 2021 and December 2023, all of whom underwent the above modified procedure. Perioperative parameters were recorded. Neurological function before and after surgery was assessed using the Glasgow Coma Scale (GCS), modified Rankin Scale (mRS), Markwalder grading system, and Glasgow Outcome Scale (GOS). Postoperative complications and hematoma recurrence were monitored during follow-up.

**Results:**

Complete hematoma evacuation was achieved in all patients, with improved midline shift and satisfactory brain re-expansion. Neurological function was improved postoperatively, as reflected by optimized GCS, mRS, Markwalder grade, and GOS scores. Postoperative complications occurred in 5 patients (29.41%), including subdural effusion, cerebrospinal fluid leak, secondary intracranial hemorrhage, and epilepsy, all of which resolved with conservative management. No recurrence was observed according to the 90-day postoperative recurrence criteria. No ipsilateral hematoma recurrence or new neurological deficits were noted during 1-year follow-up.

**Conclusion:**

In highly selected patients with recurrent organized CSDH, this modified craniotomy technique appears feasible and reasonably safe, with encouraging short-term neurological improvement. No recurrence was observed per the predefined 90-day endpoint; 1-year follow-up was descriptive. This study is a small-sample, single-center, uncontrolled retrospective case series in a highly selected cohort. The results should be interpreted as preliminary safety and feasibility data, and no assertions regarding superior efficacy or recurrence reduction can be made. Large-scale controlled studies are warranted to further validate clinical utility.

## Introduction

Chronic subdural hematoma (CSDH) is a common form of intracranial hemorrhage, defined as a collection of fluid, blood, and blood degradation products encapsulated between the arachnoid and dura mater for more than three weeks (1). Under certain mechanisms, a liquid hematoma becomes organized into an old thrombus, exhibiting multiseptated, calcified, multilobulated, or multilayered structures (2); this subtype is termed organized chronic subdural hematoma (OCSH). As a special type of CSDH, the annual incidence of OCSH ranges from 5 to 58 per 100,000 population (3). Typically, CSDH organization takes 6–12 months, whereas early organization occurring within approximately one week is rare, and most reported cases of early organization occur after soft-channel drainage (4).

Two major challenges in the surgical management of CSDH are complete relief of hematoma mass effect and prevention of postoperative recurrence (5). Currently, burr-hole drainage is the first-line surgical approach for CSDH (6), yet it is associated with a relatively high recurrence rate. Reported recurrence rates after CSDH surgery range from 2.5% to 33% (7). Although burr-hole drainage alleviates mass effect, it does not disrupt the pathophysiological mechanisms underlying CSDH, which is the primary cause of high recurrence. Furthermore, this procedure is effective mainly for liquid hematomas; organized clots cannot be evacuated through small burr holes, often requiring craniotomy for hematoma removal. This retrospective study analyzed 17 patients with recurrent organized CSDH treated at Xinghua People's Hospital between January 2021 and December 2023. Organized CSDH was diagnosed comprehensively based on prolonged clinical history, preoperative cranial CT/MRI findings suggestive of organized hematoma (e.g., septation, calcification, or multiloculation), and direct intraoperative observation of organized, septated clot during attempted burr-hole drainage. All patients underwent craniotomy for hematoma evacuation followed by middle meningeal artery occlusion and temporalis muscle grafting. The aim was to preliminarily evaluate the safety, neurological improvement, and recurrence control of this modified technique, and to summarize postoperative outcomes, with the hope of providing preliminary safety and feasibility data to inform clinical management for such patients.

### Clinical data and methods

#### General data

During the study period (January 2021–December 2023), a total of 650 patients underwent surgical treatment for chronic subdural hematoma at our institution, including 217 in 2021, 198 in 2022, and 235 in 2023. The 17 patients included in this study accounted for 2.62% of all surgically treated CSDH patients, confirming that recurrent organized CSDH is a rare and highly selected clinical subgroup. The cohort comprised 11 males and 6 females, aged 63–82 years (mean, 72.88 ± 5.73 years). The hematoma volume ranged from 50 mL to 150 mL, with a mean of (86.11 ± 25.47) mL. The hematoma thickness ranged from 8 mm to 28 mm, with a mean of (16.06 ± 4.52) mm. The preoperative midline shift ranged from 8 mm to 21 mm, with a mean of (13.27 ± 0.35) mm. Eight patients had a history of hypertension, and five had diabetes mellitus. All patients had previously undergone burr-hole drainage: 15 patients had one prior surgery, and 2 patients had two prior surgeries. Fifteen patients had unilateral hematomas, and 2 had bilateral hematomas (1 patient required bilateral surgery; 1 patient underwent unilateral surgery, with the contralateral side not meeting surgical indications) (Tables 1, 2). For bilateral cases, statistical analysis was performed on the operated side only. For the patient who underwent bilateral surgery, data from both sides were analyzed separately. For the patient with unilateral surgery and contralateral conservative management, only the operated side was included in the analysis, while follow-up data for the non-operated side were recorded but not statistically analyzed.

**Table 1:** Baseline clinical characteristics of 17 patients.

Items	Mean (Range)/Number of Cases (Percentage, %)
Age (years)	72.88（63∼82）
Gender	
Male	11（64.71）
Female	6（35.29）
History of hypertension	8（47.06）
History of diabetes mellitus	5（29.41）
Main admission symptoms	
Headache and dizziness	17（100）
Nausea and vomiting	4（23.53）
Limb hemiplegia	8（47.06）
Hemisensory disturbance	6（35.29）
Urinary and fecal incontinence	3（17.65）
Preoperative GCS score	
13–15 points	10（58.82）
9–12 points	5（29.41）
≤7 points	2（11.76）
Imaging features	
Left side	9（52.94）
Right side	6（35.29）
Bilateral sides	2（11.76）
Midline shift (mm, excluding bilateral cases)	13.27（8∼21）
Hematoma thickness (mm, excluding non-surgical side in bilateral cases)	15.89（8∼28）
Hematoma volume (mL, excluding non-surgical side in bilateral cases)	86.11（50∼150）

**Table 2:** Detailed clinical data of individual patients.

Case No.	Gender	Age (years)	Previous surgeries	Unilateral/Bilateral	Comorbidities		Midline shift (mm)	Hematoma volume (mL)	Hematoma thickness (mm)
1	Male	63	1	Unilateral	HTN	/	8	60	13
2	Male	77	1	Unilateral	HTN	DM	12	80	16
3	Male	81	1	Unilateral	HTN	/	10	70	14
4	Male	72	2	Unilateral	/	DM	15	100	17
5	Male	75	1	Bilateral	/	/	Excluded from analysis	120	23
6	Male	67	1	Unilateral	HTN	/	13	90	15
7	Male	76	1	Unilateral	/	/	12	70	16
8	Male	75	1	Unilateral	/	/	15	80	15
9	Male	65	1	Unilateral	/	/	18	110	20
10	Male	80	1	Unilateral	HTN	DM	14	90	17
11	Male	69	2	Unilateral	HTN	DM	15	100	18
12	Female	75	1	Unilateral	/	/	13	80	16
13	Female	82	1	Bilateral	HTN	/	Excluded from analysis	110	13
								60	8
14	Female	69	1	Unilateral	/	/	21	150	28
15	Female	66	1	Unilateral	/	DM	13	70	15
16	Female	76	1	Unilateral	HTN	/	11	60	12
17	Female	71	1	Unilateral	/	/	9	50	10

#### Clinical manifestations

All patients presented with varying degrees of headache and dizziness before admission. Nausea and vomiting occurred in 4 cases, hemiplegia in 8 cases, hemisensory disturbance in 6 cases, and urinary and fecal incontinence in 3 cases. According to Glasgow Coma Scale (GCS) scores, 10 patients scored 13–15 points, 5 scored 9–12 points, and 2 scored 7 points. The modified Rankin Scale (mRS) was adopted to assess neurological disability and daily living independence. One case was graded as score 1, 8 cases as score 2, 2 cases as score 3, 4 cases as score 4, and 2 cases as score 5.The Markwalder grading system was used to evaluate consciousness and neurological deficits. There was 1 case at grade I, 9 cases at grade II, 5 cases at grade III, and 2 cases at grade IV.

#### Inclusion criteria

① Diagnosis of chronic subdural hematoma confirmed by imaging examination; ② Hematoma volume and clinical symptoms met surgical indications; ③ Recurrent hematoma after previous burr-hole drainage; ④ Intraoperatively, after burr-hole creation and dural incision, organized, septated hematoma was directly confirmed, requiring conversion from planned burr-hole drainage to craniotomy; ⑤ Surgical risks were fully informed to family members, and written informed consent was obtained prior to operation. Organized CSDH was defined clinically, radiologically, and intraoperatively: prolonged clinical history, preoperative imaging demonstrating septation, calcification, or multiloculation, and intraoperative confirmation of organized clot.

This study was approved by the Ethics Committee of our hospital (Approval No. JSXHRYLL-WK-202301). All consecutive patients meeting the criteria for recurrent organized CSDH during the study period (January 2021–December 2023) were included without selective exclusion. All eligible recurrent patients received the modified surgical treatment. No selective bias existed, and no alternative therapeutic regimens were applied.

#### Exclusion criteria

① Presence of mental disorders; ② Abnormal hepatic and renal function; ③ Coagulopathy; ④ Other intracranial lesions; ⑤ Absolute contraindications to surgery; ⑥ Loss to follow-up.

### Surgical method

**Figure 1:**
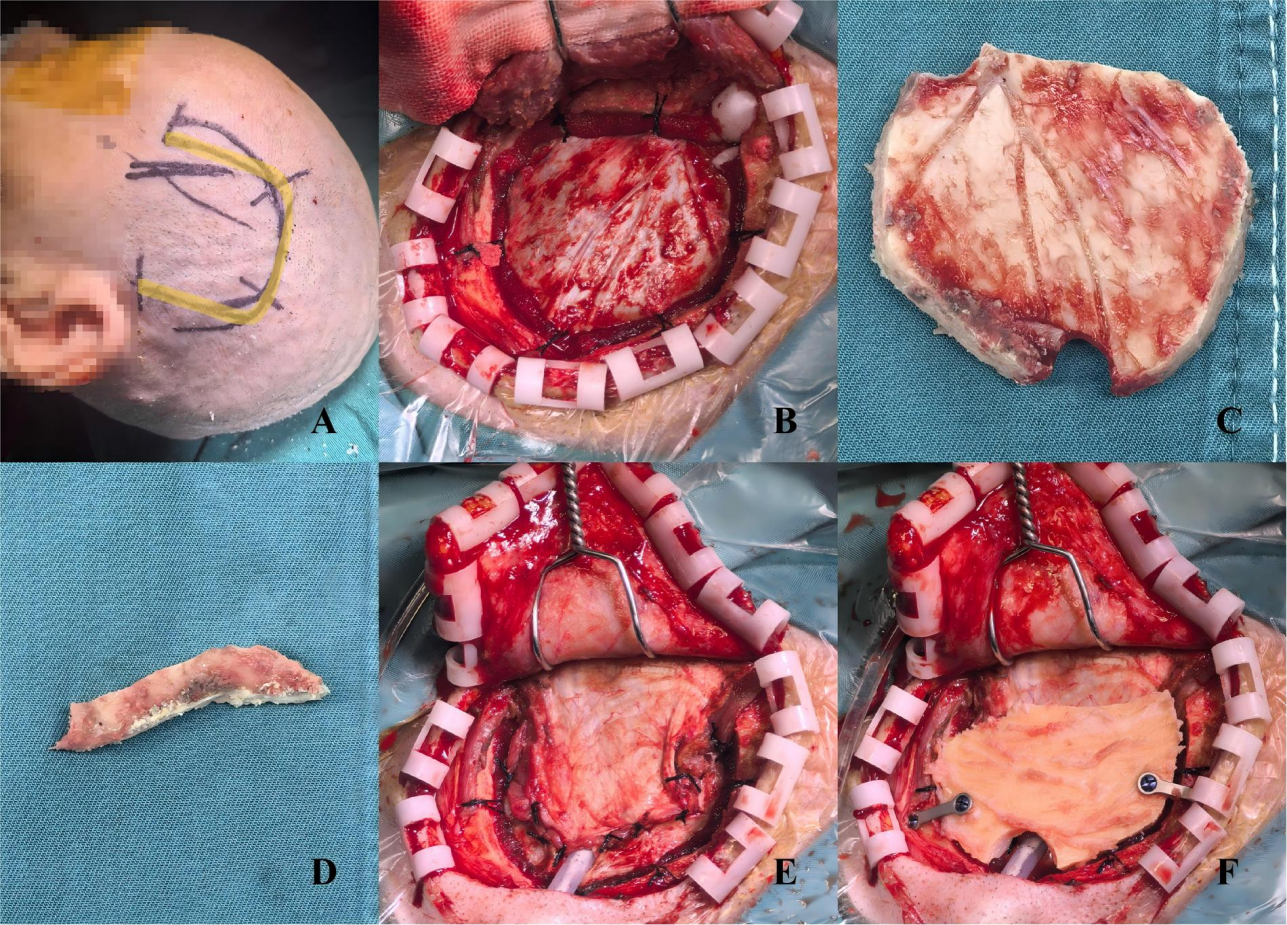
Intraoperative images of the modified surgical technique. **(A)** Preoperative skin marking of the planned incision. **(B)** Intraoperative exposure of the middle meningeal artery. **(C)** Middle meningeal artery groove on the inner bone flap surface. **(D)** A bone strip approximately 1 cm in width is resected from the inferior margin of the bone window to accommodate passage of the temporalis muscle. **(E)** Dissect the temporal muscle and suture it to the edge of the dura mater. **(F)** The bone flap is replaced, with the temporalis muscle passing beneath the flap.

A longitudinal incision of 3–4 cm was made centered on the maximum hematoma layer of cranial CT scanning. The scalp was dissected down to the periosteum, and a mastoid retractor was applied to expose the surgical field. Craniotomy was performed, and the burr hole was enlarged to approximately 1.5 cm × 2 cm using rongeurs, followed by hemostasis with bone wax and electrocoagulation. The dura mater was cauterized in a Y-shaped pattern and incised along the coagulated line. After confirmation of organized hematoma, the initial incision was extended into an 18 cm horseshoe-shaped temporoparietal incision. The skin and temporalis muscle were dissected to the skull, and the myocutaneous flap was elevated toward the skull base and fixed with scalp retractors. A bone flap of about 5 cm × 5 cm was created with a milling cutter. The exposed middle meningeal artery was thoroughly coagulated using bipolar electrocautery, and the dura mater was suspended. The dura was incised in a horseshoe shape, and the parietal layer of the hematoma membrane was resected after electrocoagulation, while the visceral layer and junctional membrane were preserved. Hematoma and organized tissue were completely aspirated. The middle meningeal artery was re-coagulated intradurally toward the skull base. No active bleeding was detected after repeated inspection. A 12-French silicone drainage tube was placed in the hematoma cavity and externally diverted through a separate scalp stab wound. The temporalis muscle was dissected and trimmed to match the craniectomy margin, then sutured to the dural edge. Artificial dura was used to repair dural defects. A small portion of the inferior bone flap was removed to accommodate the temporalis muscle, and the bone flap was repositioned and fixed with titanium plates and screws. The scalp was closed layer by layer, and the drainage tube was connected to a drainage bag (Figure 1).

The average operative duration was 90 min, with intraoperative blood loss ranging from 50 to 100 mL. Cranial CT re-examination was performed on the second postoperative day. The drainage tube was retained for 2–3 days and removed once no bloody discharge was observed. Immediate tube removal was conducted if massive cerebrospinal fluid leakage occurred due to membrane rupture. Patients were hospitalized for another 2–3 days after tube removal and received final cranial CT scanning before discharge. The overall hospital stay lasted approximately one week.

### Follow-up protocol

Routine follow-up was conducted at 1, 3, 6 and 12 months postoperatively, with cranial CT examination and clinical assessment including neurological function, symptoms and signs performed at each visit. Recurrence was strictly defined as radiologically confirmed re-enlargement of the operated-side hematoma or neurological deterioration requiring reoperation within 90 days postoperatively (8). Residual hematoma, asymptomatic subdural effusion, and asymptomatic hematoma accumulation were not considered recurrence. The 12-month follow-up was descriptive, intended only for observing long-term neurological status and hematoma stability, and was not used for recurrence assessment.

### Statistical analysis

This was a small-sample case series analyzed by descriptive statistics. Measurement data were presented as mean ± standard deviation (x ± s）, and enumeration data were expressed as case number and percentage. No inferential statistical tests were performed. For patients with bilateral hematomas, statistical analysis was conducted based on the operated side, while the non-operated side was excluded from primary outcome assessment.

## Results

### Postoperative outcomes

Complete hematoma evacuation was achieved in all patients. The postoperative midline shift recovered to (5.27 ± 1.49) mm, and the brain re-expansion rate reached (82.3 ± 4.5)%. Headache and dizziness were relieved to varying degrees in all cases. Nausea and vomiting disappeared in the 4 affected patients. Among 8 patients with hemiplegia, muscle strength fully recovered in 6 cases; the remaining 2 cases presented improved muscle strength without reaching grade V, which was attributed to insufficient brain re-expansion and concurrent postoperative subdural effusion. Of 6 patients with hemisensory disturbance, symptoms resolved completely in 5 cases and partially improved in 1 case.

### Neurological function recovery

Postoperative GCS scores: 14 cases scored 15 points, 2 cases scored 14 points, and 1 case scored 13 points. Postoperative mRS scores included 9 cases of grade 0, 6 cases of grade 1 and 2 cases of grade 2. Postoperative Markwalder grading showed 11 cases of grade 0 and 6 cases of grade I. GOS scores were 5 points in 14 cases and 4 points in 3 cases.

### Postoperative complications

**Figure 2:**
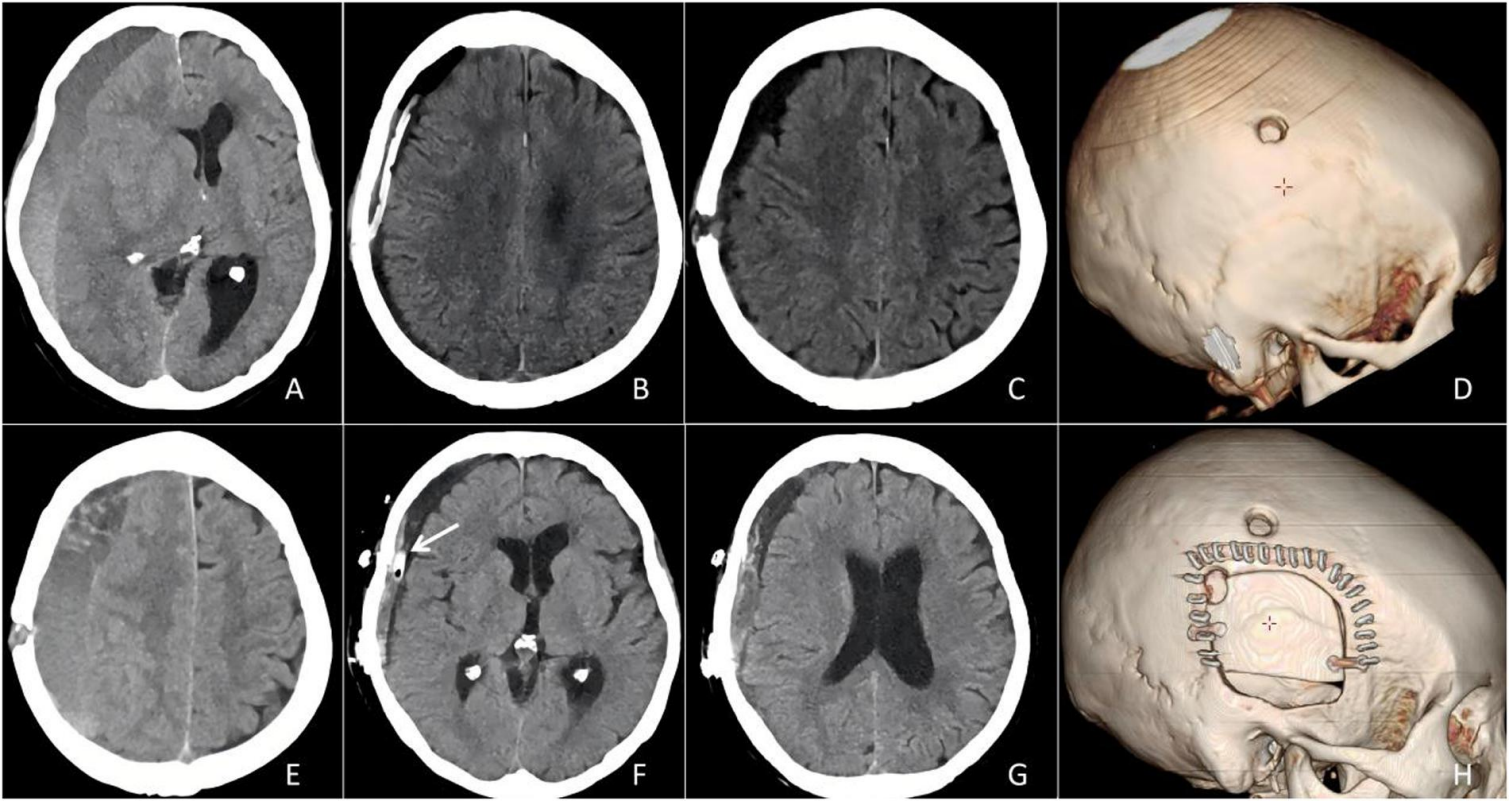
Preoperative and postoperative cranial CT scans in a representative patient. **(A)** Initial preoperative chronic subdural hematoma. **(B)** Postoperative day 2 CT after first burr-hole drainage. **(C)** Pre-discharge CT after first surgery. **(D)** Burr-hole site from the first procedure. **(E)** Hematoma recurrence at 2 months postoperatively. **(F)** Postoperative day 1 CT after modified craniotomy (arrow: drainage tube). **(G)** Cranial CT obtained four days after the second surgery, prior to discharge; the increased density beneath the bone flap corresponds to the applied temporalis muscle. **(H)** Bone flap replacement during the second surgery; the bony defect beneath the flap was created intraoperatively using a milling cutter to allow passage of the temporalis muscle.

A total of 5 postoperative complications occurred among 17 patients, including 2 cases (11.76%) of subdural effusion, 1 case (5.88%) of cerebrospinal fluid leakage, 1 case (5.88%) of secondary intracranial hemorrhage, and 1 case (5.88%) of epilepsy. No tension pneumocephalus or intracranial infection was found. The hematoma was gradually absorbed after conservative treatment in patients with secondary intracranial hemorrhage. Hyperbaric oxygen therapy was administered for subdural effusion, resulting in brain tissue expansion and gradual resolution of effusion. Epileptic symptoms were well controlled by oral medication (Table 3, Figure 2).

**Table 3:** Postoperative complications and outcomes.

Postoperative Outcomes	Number of Cases (Percentage, %)
Subdural effusion	2（11.76）
Cerebrospinal fluid (CSF) leakage	1（5.88）
Secondary intracranial hemorrhage	1（5.88）
Epilepsy	1（5.88）
Tension pneumocephalus	0（0）
Intracranial infection	0（0）
Postoperative recurrence	0（0）

### Hematoma recurrence

Among the 17 patients, 2 suffered from bilateral hematomas. One patient received unilateral surgery while the contralateral hematoma did not meet surgical criteria and was gradually absorbed after oral administration of atorvastatin calcium. All patients underwent cranial CT scanning and clinical assessment at 1, 3, 6 and 12 months postoperatively. No recurrence was identified per the predefined 90-day endpoint. The 1-year follow-up remained descriptive, with no delayed hematoma recurrence or new neurological deficits detected.

## Discussion

Chronic subdural hematoma (CSDH) is a common hemorrhagic neurological disease, accounting for approximately 10% of hemorrhagic stroke cases (9). Accumulation of fluid, blood and blood degradation products in the subdural space leads to chronic space-occupying lesions. Chronic subdural hemorrhage triggers local inflammatory responses and releases plasminogen. Activated plasminogen converts into plasmin, which accelerates fibrinolysis, inhibits platelet aggregation and causes persistent chronic bleeding (10). Massive flocculent substances generated from fibrin degradation also serve as a risk factor for hematoma recurrence (11). Histopathological studies have verified neovascular capillaries on the hematoma membrane. Widened endothelial gaps elevate vascular permeability and fragility, resulting in continuous blood leakage (12). Further research confirms that these neovessels are supplied by the middle meningeal artery (MMA). Superselective cerebral angiography reveals dilated MMA and cotton-like vascular networks at the distal segment, indicating microvascular oozing (13). Accordingly, clinical treatment should not only evacuate liquid hematoma and relieve mass effect, but also thoroughly remove fibrin floccules and hematoma membrane to mitigate local inflammation (14). Blocking MMA perfusion to neovascular clusters on the hematoma membrane can effectively prevent CSDH recurrence (15).

CSDH treatments are divided into conservative and surgical modalities. Conservative therapy mainly consists of oral atorvastatin and hormones. As the only drug with level I evidence for CSDH treatment, atorvastatin is promising to become a first-line therapeutic option. It exerts anti-angiogenic effects via inhibiting vascular endothelial growth factor and interleukin-8, anti-inflammatory effects by reducing tumor necrosis factor-α and monocyte chemoattractant protein-1, and anti-fibrotic activity through lowering collagen deposition. Nevertheless, 11.2% of patients fail to respond to medication and eventually require surgery (16). In this study, one patient with bilateral hematoma received craniotomy on one side, while the contralateral hematoma resolved gradually after oral atorvastatin administration, with no recurrence observed during one-year follow-up. The drug mainly suppresses intracavity inflammation and persistent membrane bleeding, which targets the pathogenesis of CSDH and yields reliable clinical efficacy.

Surgical treatments mainly include burr-hole drainage, middle meningeal artery embolization (MMAE), craniotomy hematoma evacuation and small-window endoscopic hematoma removal. Burr-hole drainage serves as the primary surgical option for CSDH due to its safety, minimal trauma and adequate drainage capacity. However, blind catheter placement limits thorough intracavitary irrigation. It fails to break through septa in organized and compartmentalized hematomas, potentially elevating recurrence rate up to 33% (17). Reoperation carries high risks and poor prognosis for repeatedly relapsed patients, who are usually elderly with multiple comorbidities and coagulation disorders (18). Pharmacotherapy targeting inflammation and angiogenesis also shows limited efficacy (19). To reduce recurrence and improve therapeutic outcomes, MMAE has been increasingly applied in CSDH management in recent years (20–22). It blocks blood supply to the outer hematoma membrane via arterial embolization, alleviating blood exudation and facilitating hematoma resolution, with advantages of safety, minimal invasiveness and low recurrence risk (23, 24). In 1999, Tanaka et al. (25) first identified the correlation between MMA and vascular supply of hematoma membrane. Subsequent studies confirmed ipsilateral MMA dilation compared with the contralateral side and the vessel caliber prior to onset (26). The first refractory CSDH case was successfully treated with MMAE in 2000, and multiple clinical trials have validated its safety and efficacy thereafter (27, 28). Combined burr-hole drainage and MMAE accelerates hematoma absorption (29). MMAE alone or in combination offers a novel therapeutic strategy for recurrent and refractory CSDH. Relevant research reports a technical success rate of 97.4% and a 90-day reoperation rate of 6.5%, with remarkable hematoma resolution in most cases (30). Comparative studies demonstrate equivalent efficacy between standalone MMAE and combined therapy for recurrent CSDH, with superior minimally invasive merits of single MMAE (31).Craniotomy is reserved for highly organized, severely adhered and recurrent hematomas. It provides sufficient surgical exposure for membrane and septum resection and thorough hemostasis (32), yielding the lowest recurrence rate yet accompanied by high operative risks, severe tissue injury and frequent complications, thus restricting its clinical application (33). Burr-hole drainage barely works for organized hematomas eligible for surgery, as organized tissue cannot be drained through small holes, necessitating craniotomy evacuation.All 17 enrolled patients suffered from recurrent organized CSDH, and two had undergone two prior drainage procedures. Although MMAE could mitigate relapse risks, concurrent mass compression still required secondary craniotomy, imposing heavy psychological and economic burdens on patients. Therefore, targeted at inflammation and persistent bleeding from the parietal membrane, we conducted tentative intraoperative intervention during craniotomy for organized hematoma evacuation.

A temporoparietal horseshoe incision was adopted in this study. Compared with conventional straight temporal incision, this approach possesses distinct advantages. It enables adequate exposure and dissection of the temporalis muscle to expand the attachment area. Rich blood perfusion and masticatory movement of temporalis muscle may absorb residual inflammatory factors and fibrin degradation products, breaking the vicious cycle of hematoma enlargement. This temporally centered incision fully exposes the trunk and branches of middle meningeal artery. Electrocautery occlusion of the whole arterial course inside and outside the dura after hematoma removal cuts off blood supply to hematoma membrane and reduces continuous bleeding, exerting similar therapeutic effect as MMAE in preventing recurrence.Moreover, bone flap replacement can be realized in this operation. Traditional burr-hole procedures fail to restore skull integrity, and enlarged bone windows over 3 cm usually meet the surgical indication for cranioplasty, which may cause local skull depression, unstable intracranial pressure and potential cerebral injury. The skull defect in this study is merely about 1.5 cm at the temporal base, designed for temporalis muscle placement, with no cosmetic deformity, intracranial pressure fluctuation or cerebral damage risk.

With updated therapeutic concepts for chronic subdural hematoma, multiple novel surgical strategies have been applied to prevent recurrence, such as membrane management, endoscopic hematoma evacuation, normal saline irrigation and modified closed drainage. Resection of parietal membrane has been attempted for refractory CSDH to reduce persistent oozing, yet it fails to fundamentally cut off blood supply. Complete membrane removal is hard to achieve, and residual membrane tends to bleed again, triggering secondary hemorrhage and hematoma relapse that may require emergency craniotomy. Endoscope-assisted small-window craniotomy combined with middle meningeal artery occlusion has also been practiced. This method cannot guarantee thorough arterial occlusion, since the main trunk runs along the outer dural surface adhering to the skull (34), making accurate visualization and complete occlusion difficult from the intradural side. Recent studies on controllable recurrence risk factors identify postoperative intracranial pneumocephalus as a major hazard. A large-sample study involving 460 patients by Scala et al. (35) verified that closed drainage markedly reduces pneumocephalus volume, recurrence rate and hospital stay compared with conventional burr-hole irrigation and drainage. Minimizing intraoperative air entry and stabilizing intracranial pressure are critical for relapse prevention. Reducing hematoma recurrence remains a core challenge in CSDH treatment.

Among the 17 patients, 5 developed postoperative complications, with an overall complication rate of 29.41%, including 2 cases (11.76%) of subdural effusion, 1 case (5.88%) of cerebrospinal fluid leakage, 1 case (5.88%) of secondary intracranial hemorrhage, and 1 case (5.88%) of epilepsy. Although all complications resolved with conservative management, this relatively high rate warrants prudent interpretation and explicit risk–benefit assessment. These events, including cerebrospinal fluid leakage and secondary hemorrhage, are not negligible and reflect the inherent invasiveness of craniotomy for complex, recurrent organized CSDH. Therefore, the potential benefits of this modified technique—namely reliable hematoma evacuation and favorable short-term recurrence control—must be carefully weighed against this non-negligible complication risk in clinical decision-making. Temporalis muscle attachment is widely applied in neurosurgical operations for massive cerebral infarction, moyamoya disease and subdural effusion. Traditional approaches inevitably lead to bone flap removal and skull defect. In this study, the temporalis muscle was sutured to the dura mater with reserved space beneath the bone flap for muscle passage, enabling bone flap replacement. Concerns over discomfort during speaking and mastication as well as cosmetic appearance were raised beforehand, yet no related complaints were found during follow-up. This may be attributed to age-related temporalis muscle atrophy and hypofunction in elderly participants, as well as the limited sample size. Further long-term follow-up and expanded sample enrollment will be conducted to draw more reliable conclusions. Notably, the 90-day recurrence was predefined as the primary endpoint, whereas 1-year outcomes were descriptive and not powered for formal statistical comparison.

## Limitations

Several limitations should be acknowledged. First, the sample size is small (*n* = 17), and the study is single-center, retrospective, and uncontrolled without a comparison group. The cohort represents a highly selected population: only patients with recurrent organized CSDH who failed prior burr-hole drainage and required craniotomy were included. Accordingly, this work should be framed as a safety and feasibility report, not as an efficacy trial. We cannot assert that the technique reduces recurrence or outperforms alternative surgical methods. Second, follow-up was limited to 1 year, and long-term recurrence and safety remain unknown. Third, the relatively high postoperative complication rate (29.41%) is a notable concern; although all complications resolved with conservative management, they reflect the invasiveness of craniotomy for complex recurrent organized CSDH, requiring careful risk–benefit assessment. Fourth, although strict diagnostic criteria were applied and all consecutive eligible patients were enrolled, selection bias cannot be fully excluded. The observed absence of 90-day recurrence is a preliminary finding that needs validation in larger controlled studies.

## Conclusion

This small retrospective series included 17 highly selected patients with recurrent organized CSDH who had failed prior burr-hole drainage. The modified craniotomy approach—combining hematoma evacuation, middle meningeal artery occlusion, temporalis muscle grafting, and bone flap replacement—appears feasible and reasonably safe, with encouraging short-term neurological outcomes. No recurrence was observed per the predefined 90-day endpoint; 1-year follow-up was descriptive. This report is limited to safety and feasibility in a highly selected cohort, and no claims of superior efficacy or recurrence benefit are made. Larger controlled studies are essential to confirm clinical utility and comparative performance.

## Data Availability

The original contributions presented in the study are included in the article/Supplementary Material, further inquiries can be directed to the corresponding author.
